# Case Report of Customized Distal Radius Prosthesis Replacement: An Alternative Treatment for Post-traumatic Unreconstructable Intraarticular Distal Radius Malunion

**DOI:** 10.7759/cureus.7841

**Published:** 2020-04-26

**Authors:** Woraphon Jaroenporn, Jaruwat Vechasilp, Pradit Predeeprompan, Sunyarn Niempoog, Jirantanin Rattanavarinchai

**Affiliations:** 1 Hand and Microsurgery, Orthopaedic Surgery, Police General Hospital, Bangkok, THA; 2 Orthopaedics, Police General Hospital, Bangkok, THA; 3 Orthopaedics, Thammasat University, Bangkok, THA

**Keywords:** distal radius malunion, unreconstructable, arthroplasty, distal radius, motion preservation, hemiarthroplasty

## Abstract

Severely comminuted intraarticular distal radius malunion can significantly affect a patient's quality of life. To date, there is no ideal solution. We propose customized distal radius prosthesis replacement as a treatment option. A 33-year-old policeman presented with left wrist deformity and loss of motion for five months following a distal radius fracture AO (Arbeitsgemeinschaft für Osteosynthesefragen) type-C3 which had been fixed with a volar locking plate incorporate with external fixation and Kirschner wire (K-wire) augmentation for two months. He needed to rely on wrist motion for work. Therefore, we fabricated a customized distal radius prosthesis based on his contralateral normal anatomy to replace the malunion site. The patient was satisfied and able to return to work two months after the operation. Thirty months later, the range of motion had improved from fixed 40° flexion and fixed 70° pronation deformity to 73° flexion, 79° extension, 75° supination, and 85° pronation. His DASH (Disabilities of the Arm, Shoulder, and Hand) score had improved from 80 to 14.2. His pain score, as measured by the visual analog scale, improved from eight preoperatively to two. Unreconstructable intraarticular malunion of the distal radius is a challenging problem with no treatment consensus. Customized distal radius prosthesis may provide a successful treatment option. Future research should elucidate long-term outcomes.

## Introduction

Distal radius fracture represents 20% of adult fractures with the intraarticular component accounting for as much as 50% of cases [1]. Without achieving anatomic reduction, malunion alters contact stresses resulting in posttraumatic arthritis [2]. This can result in significant deterioration of patient quality of life. Historically, wrist arthrodesis has been the mainstay treatment for painful osteoarthritis of the wrist [3]. Even though grip strength and pain relief are predictable, the loss of joint mobility is a major functional complication. Total wrist arthroplasty (TWA) and partial wrist arthroplasty were developed to maintain wrist motion as well as relieve pain. Despite advancements in total wrist implant design and surgical technique, complications are often devastating [4-6]. With distal component loosening as the major cause for revision, distal radius hemiarthroplasty (DRH) has been proposed [7]. Overall outcomes suggest that DRH is susceptible to the same set of complications as TWA, many of which are related to polyethylene disease [8]. Intending to achieve a painless wrist function, we designed a customized distal radius prosthesis (DRP) based on the patient's normal contralateral radius anatomy. To create perfect joint congruency, the DRP was fabricated individually to best match the patient's carpal bone anatomy, creating a maximal contact area and minimizing bone loss.

## Case presentation

**Figure 1:**
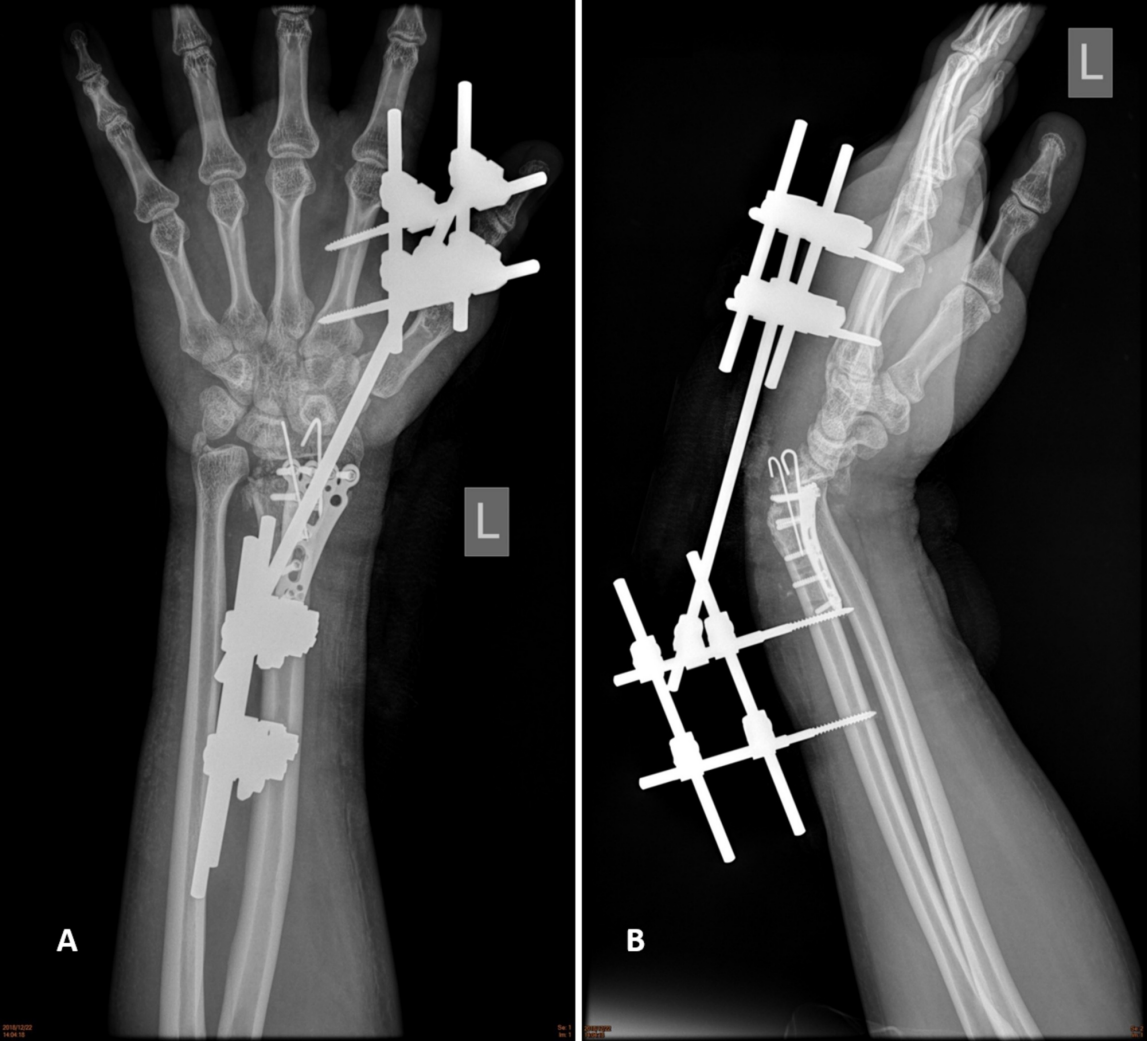
Preoperative film. (A) Anteroposterior. (B) Lateral.

A 33-year-old right-hand dominant Thai policeman presented at Police General Hospital with left wrist pain and fixed deformity. He was diagnosed with left severely comminuted distal radius fracture, AO (Arbeitsgemeinschaft für Osteosynthesefragen) type-C3, and was treated with a volar locking plate augmented with an external fixator and Kirshner wires (K-wires). Two months later, the external fixator and K-wires were removed. The patient still could not move his wrist despite three months of rehabilitation and could not return to his routine activities. On physical examination, the wrist was fixed in 40° flexion and 70° pronation with distal radial ulnar joint (DRUJ) instability. On X-ray, the carpal bones were found to be translated (Figure 1). The patient emphasized the importance of regaining his hand motion in order to return to work. After discussing possible treatment options, the patient decided to proceed with distal radius prosthesis replacement.

Prosthesis design and fabrication

**Figure 2:**
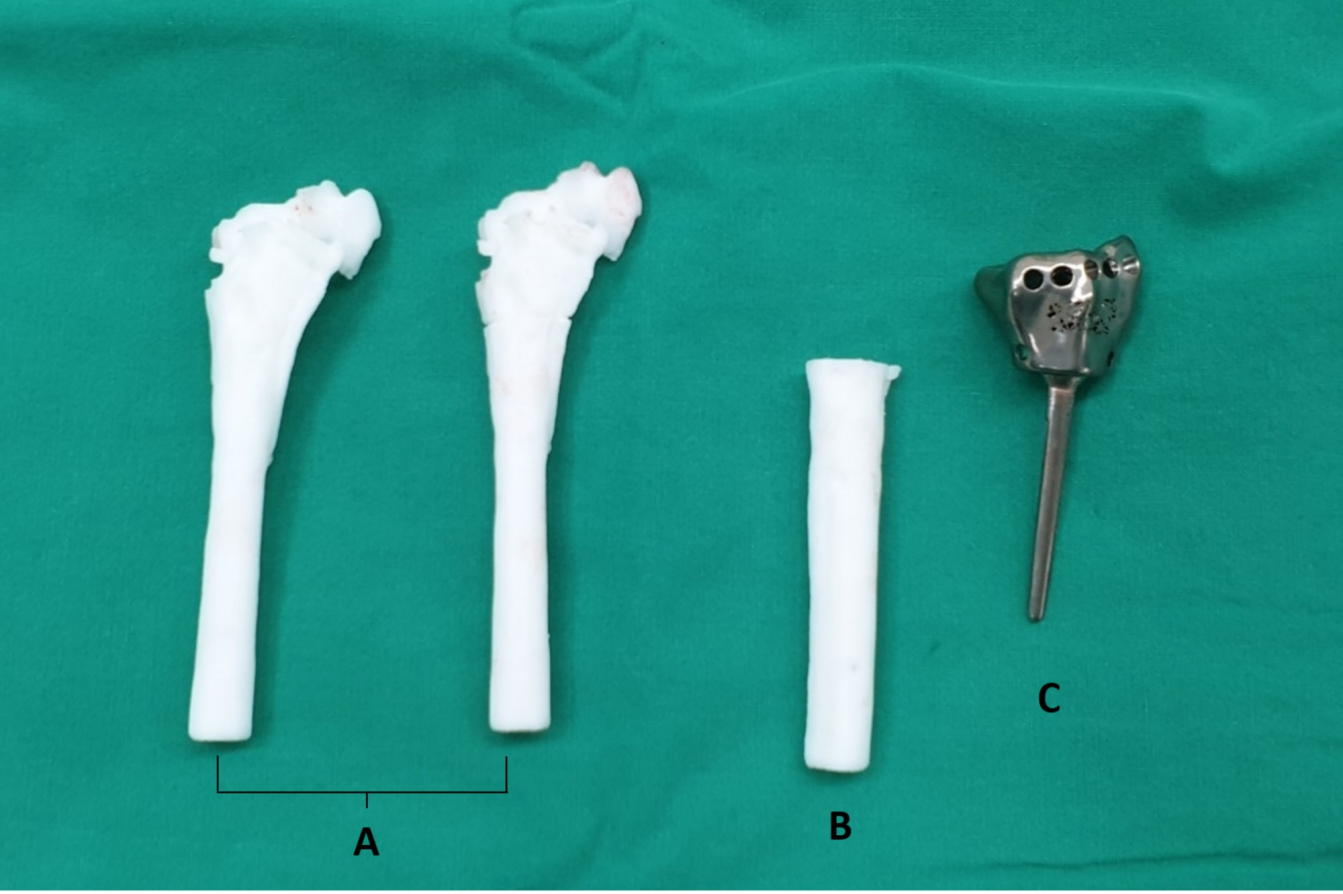
Customized distal radius prosthesis and cutting guide. (A) Patient’s malunion model. (B) Cutting guide. (C) Distal radius prosthesis.

A computed tomography scan of the patient's bilateral wrists was scheduled per protocol, then sent to Meticuly Co., Ltd., Thailand to fabricate the distal radius prosthesis (full metal) and cutting guide designed by our clinical team based on the patient's normal contralateral distal radius (Figure 2).

Surgical technique

**Figure 3:**
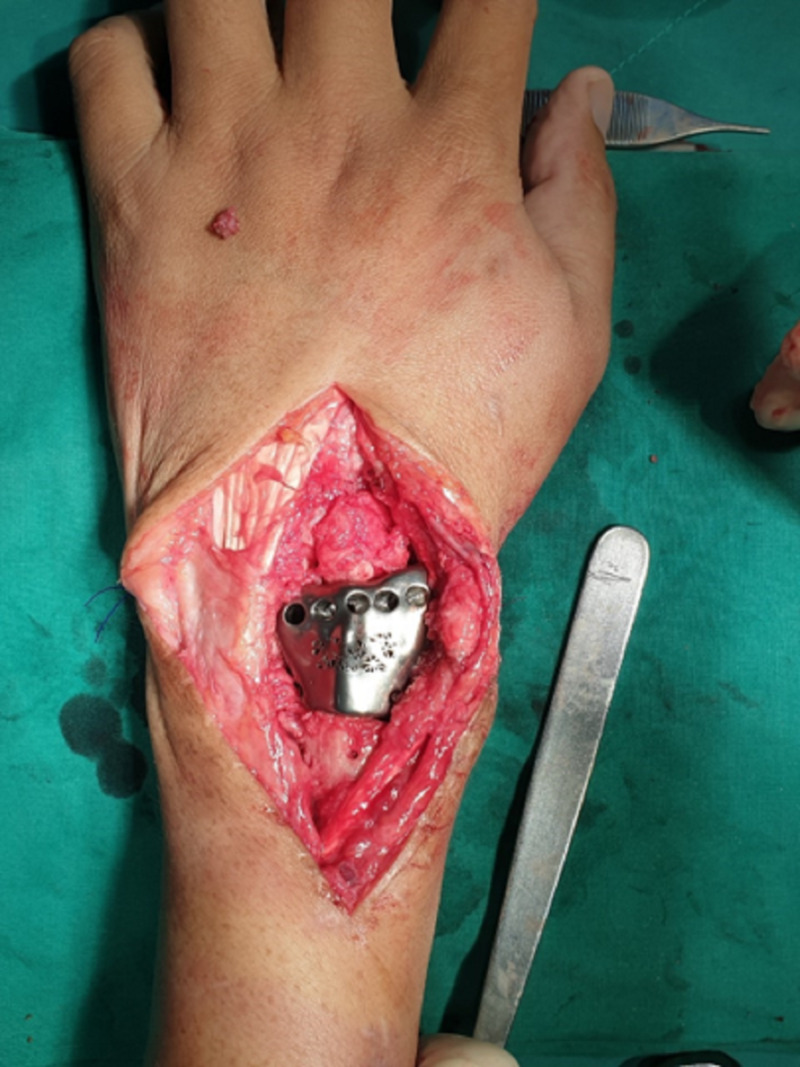
Placement of the customized distal radius prosthesis

Under general anesthesia, the patient was placed in the supine position. First, the volar approach was used to remove the volar locking plate though the patient's old surgical scar. A longitudinal dorsal approach though the fourth compartment was used to identify the malunion site. The customized cutting guide was used to determine the osteotomy site. The cartilage on the lunate was well preserved. The malunion site was excised with caution to preserve ligaments to the carpal site as much as possible. In this case, all the radiocarpal ligaments were constricted and blended into fibrosis. The prosthesis was placed on the stump and fixed with a cemented technique. Both palmaris longus were harvested to reconstruct the dorsal radiocarpal ligament, radioscaphocapitate ligament with anchor sutures, and distal radioulnar ligament using a technique previously described by Jones and Sanders (Figure 3) [9]. Stability and motion were tested under direct vision. All the remaining soft tissue, preserved ligaments, joint capsule, and extensor retinaculum were sewed to the prosthesis pores with nonabsorbable suture to enhance joint stability.

Postoperative management

After the wrist joint underwent massive resection of its stabilizers and ligament reconstruction, it was immobilized in an above-elbow cast for four weeks to promote scarring in order to enhance joint stability. The patient was then instructed to perform active-assisted range of motion exercises for four weeks. The patient was instructed to avoid forceful activity or lifting weights heavier than 5 kg.

Results

**Figure 4:**
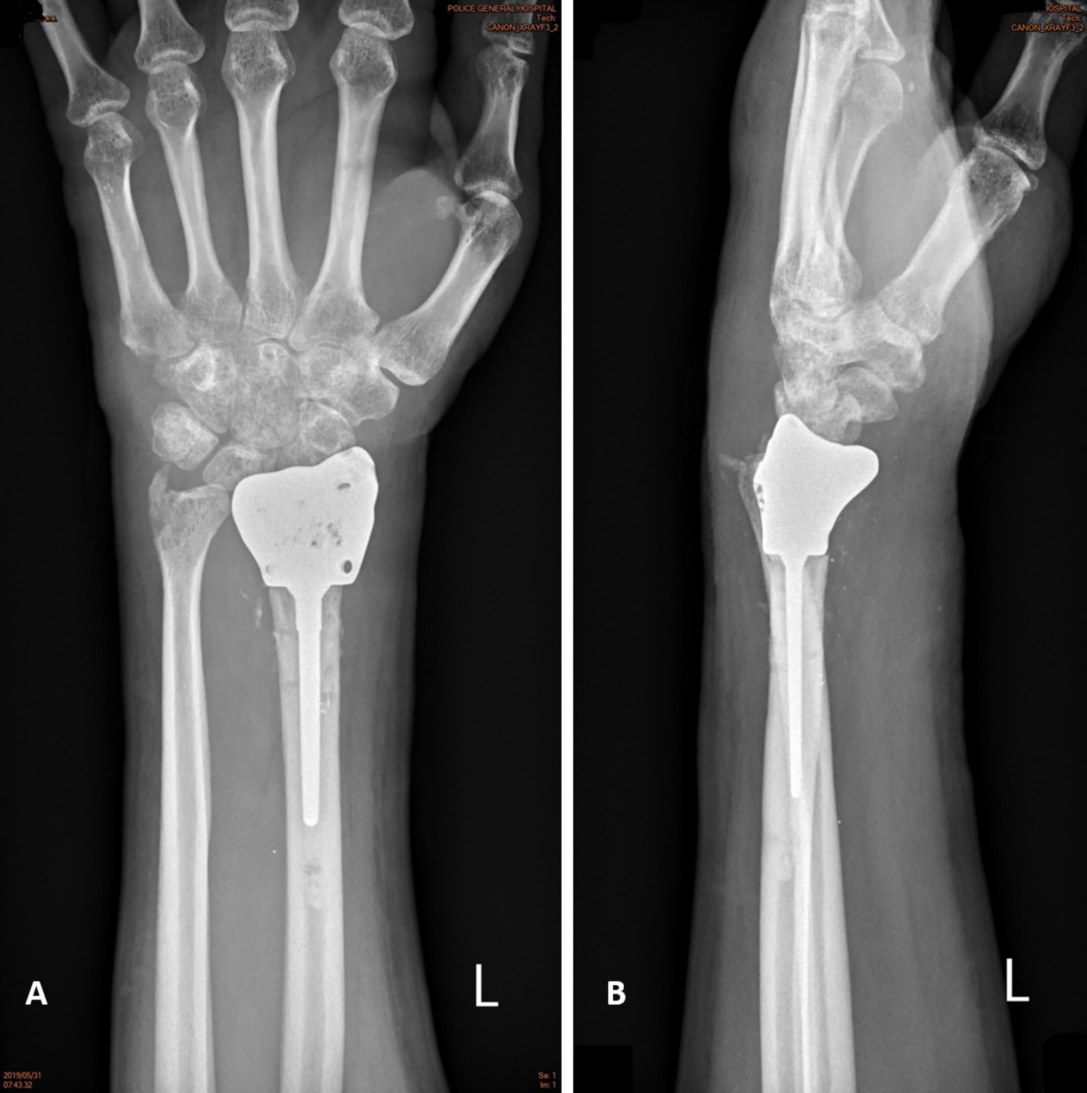
Film at thirty postoperative months. (A) Anteroposterior. (B) Lateral.

Two months after surgery, the patient was able to resume his daily activities and return to work. Thirty months after surgery, range of motion had improved from fixed 40° flexion and fixed 70° pronation to 73° flexion, 79° extension, 85° pronation, and 75° supination. His DASH (Disabilities of the Arm, Shoulder, and Hand) score prior to surgery was 80. His visual analog scale score for pain was one to two at the end of motion compared to eight preoperatively. His DASH score was 14.2. A plain X-ray showed no sign of instability or an unacceptable alignment (Figure 4). The patient reported satisfactory wrist function and said that he would choose the same treatment again if faced with a similar situation.

## Discussion

Unreconstructable intraarticular distal radius malunion can lead to severe pain and functional impairment, which result in significant deterioration of patient quality of life. Aiming to achieve a painless, functional range of motion of the wrist, many treatments have been proposed based on timing, osteoarthritis involvement, malunion configuration, and patients' functional requirements [1,10,11]. Re-cutting the displaced fractures and reducing them anatomically is one of the proposed options. Theoretically, this procedure provides an anatomic reduction of the articular surface. It can be performed with either open (outside-in) technique which provides broad exposure, or arthroscopic (inside-out) technique, developed to maintain blood supply and better visualization. However, the drawback is that the procedure should be performed within six to eight weeks from the fracture or the mature bone with filled up instead of loose granulation and scar [10]. Unfortunately, as in this case, patients often present in a delayed fashion.

Previously, wrist arthrodesis was the treatment of choice for painful arthritis [3]. Despite its predictable outcome and durability, it comes with the significant cost of loss of motion [12]. Partial arthrodesis (such as radioscapholunate fusion) provided an alternative in this scenario. However, the nonunion rate associated with this technique has been reported at 3% to 26%, which can lead to mid-carpal arthritis [12]. With knowledge and technological advancement, arthroplasty could be considered one of the treatments of choice. The principle of arthroplasty is that it preserves the functional range of motion along with joint stability. One systematic review showed that TWA is associated with a 25% incidence of major complications [13]. Carpal component loosening has been reported as the major cause of revision [14]. This is usually due to changes in kinematics and patient activity that exceed the capacity of the implants. Consequently, DRH was proposed and found to have similar complications to TWA due to polyethylene disease [8]. The customized DRP used in this case is based on the principle of hemiarthroplasty that preserves motion while trying to lower the rate of revision due to distal component loosening. The prosthesis was made of full metal material to decrease the potential for polyethylene disease. A drawback of this design is that the stability of the wrist joint depends solely on the soft tissue. As we tried to repair the ligament anatomically, all radiocarpal ligaments were found to be torn and blended into a fibrotic scar. We needed to use tendon grafts to reconstruct dorsal radiocarpal ligaments and distal radioulnar ligaments atomically and sew the soft tissue around the joint to the prosthesis's pores to enhance stability. In the 36-month follow-up report of a young patient with malunion of the distal radius treated with hemiarthroplasty and DRUJ arthroplasty, flexion/extension arc of 60°, pronation 90°, and supination 80° were documented [1]. A report of hemiarthroplasty in an elderly patient with unreconstructable distal radius fracture showed 25° flexion, 37° extension, 80° supination, and 70° pronation at 28 months postoperative follow-up [11]. Even though our reported outcomes fall in the short- to intermediate-term of 30 months, they showed excellent stability, range of motion, and clinical function. Longer-term data are needed to evaluate the durability of the prosthesis.

## Conclusions

Severe intraarticular malunion can cause severe pain and functional impairment, which often lead to significant deterioration of quality of life. Even though there are many alternative treatments, there is still no satisfactory method for restoring young patients' wrist function. We designed a customized distal radius prosthesis based on the patient's anatomy, with short-term results noninferior to other treatments. We conclude that a customized distal radius prosthesis may prove to be an alternative treatment option for severe intraarticular malunion in the young active patient. Long-term results remain to be studied. Treatment of unreconstructable intraarticular malunion of the distal radius can be challenging, especially in active young patients. Total wrist arthrodesis is still considered mainstay therapy with a predictable outcome. With further technological advancement, customized distal radius prosthesis might prove to be a good alternative treatment for the preservation of wrist range of motion and an overall satisfactory outcome.
